# Blood donor variability is a modulatory factor for *P. falciparum* invasion phenotyping assays

**DOI:** 10.1038/s41598-021-86438-1

**Published:** 2021-03-29

**Authors:** Laty G. Thiam, Prince B. Nyarko, Kwadwo A. Kusi, Makhtar Niang, Yaw Aniweh, Gordon A. Awandare

**Affiliations:** 1 West African Centre for Cell Biology of Infectious Pathogens, College of Basic and Applied Sciences, University of Ghana, Legon, Ghana; 2 Department of Biochemistry Cell and Molecular Biology, College of Basic and Applied Sciences, University of Ghana, Legon, Ghana; 3 Department of Immunology, Noguchi Memorial Institute for Medical Research, University of Ghana, Legon, Ghana; 4 Pôle Immunophysiopathologie et Maladies Infectieuses, Institut Pasteur de Dakar, Dakar, Senegal; 5 Present Address: G4 MEGA Vaccines, Institut Pasteur de Dakar, Dakar, Senegal; 6 Present Address: Laboratory of Pathogen-Host Interaction, UMR5235, CNRS, University of Montpellier, Montpellier, France

**Keywords:** Haematological diseases, Infectious diseases, Cellular microbiology, Parasitology, Pathogens, Biological techniques, Cell biology, Microbiology, Diseases

## Abstract

Human erythrocytes are indispensable for *Plasmodium falciparum* development. Unlike other eukaryotic cells, there is no existing erythroid cell line capable of supporting long-term *P. falciparum *in vitro experiments. Consequently, invasion phenotyping experiments rely on erythrocytes of different individuals. However, the contribution of the erythrocytes variation in influencing invasion rates remains unknown, which represents a challenge for conducting large-scale comparative studies. Here, we used erythrocytes of different blood groups harboring different hemoglobin genotypes to assess the relative contribution of blood donor variability in *P. falciparum* invasion phenotyping assays. For each donor, we investigated the relationship between parasite invasion phenotypes and erythrocyte phenotypic characteristics, including the expression levels of surface receptors (e.g. the human glycophorins A and C, the complement receptor 1 and decay accelerating factor), blood groups (e.g. ABO/Rh system), and hemoglobin genotypes (e.g. AA, AS and AC). Across all donors, there were significant differences in invasion efficiency following treatment with either neuraminidase, trypsin or chymotrypsin relative to the control erythrocytes. Primarily, we showed that the levels of key erythrocyte surface receptors and their sensitivity to enzyme treatment significantly differed across donors. However, invasion efficiency did not correlate with susceptibility to enzyme treatment or with the levels of the selected erythrocyte surface receptors. Furthermore, we found no relationship between *P. falciparum* invasion phenotype and blood group or hemoglobin genotype. Altogether, our findings demonstrate the need to consider erythrocyte donor uniformity and anticipate challenges associated with blood donor variability in early stages of large-scale study design.

Malaria continues to be a global public health burden, causing over two hundred million cases annually and accounting for hundreds of thousands of deaths every year^1^. Alone, *Plasmodium falciparum* accounts for more than 90% of the malaria-related mortality globally, primarily occurring in children and pregnant women living in sub-Saharan Africa^2^. Malaria-associated pathologies only manifest during the blood stage of the parasite’s life cycle. This stage is characterized by repeated rounds of asexual replications within the host erythrocyte, following the parasite’s egress from the hepatocytes. *P. falciparum* merozoites have the sole purpose to invade erythrocytes and perpetuate the asexual multiplication^3^. Given their importance in the parasite’s successful invasion and further multiplication within the host cell, merozoite antigens, and particularly invasion-related antigens represent attractive blood-stage vaccine targets. Thus, unravelling the nature of ligand-receptor interactions involved in erythrocyte invasion is essential for malaria vaccine development.

Although recent studies have enabled considerable progress in our understanding of the molecular basis of erythrocyte invasion by *Plasmodium* parasites^4,5^, little is known about the actual contribution of the host cell. Whereas there is clarity on the redundancy of ligand-receptor interactions involved in invasion, the functional relevance of some of these interactions are uncertain. Such interactions are presumed to be involved in signal transduction on either side between the parasite and the host erythrocyte^6,7^. However, pioneering reports on the major invasion profiles of *P. falciparum* clinical isolates across various malaria endemic countries have led to the hypothesis that *P. falciparum* invasion profiles are driven by the intensity of ongoing transmission in any given area^8–16^. This proposition has been challenged by recent findings, which have shown no relationship between endemicity and invasion profile when parasites from countries of varying endemicity were subjected to similar protocols^16^. This emphasizes that conducting large-scale *P. falciparum* phenotyping studies may inevitably require standardized protocols to allow comparisons across sites. One of the major drawbacks that may preclude the design of such assays is the lack of consistency in the usage of donor erythrocytes^17^. Human erythrocyte polymorphisms have been shown to be associated with the distribution of *P. falciparum* globally^18,19^. This heterogeneity may account for the differences in the reported invasion profiles using erythrocytes of different origins.

In addition, despite the progress made in generating immortalized erythroid cell-lines retaining a mature phenotype, upscaling the production of these cells for universal usage is challenging^20–25^. It is therefore of utmost importance to investigate the contribution of variation in donor erythrocytes in characterizing *P. falciparum* phenotypic diversity. Here, we present results from investigations aimed at assessing the relative contribution of blood donor variability in *P. falciparum* invasion phenotyping assays (IPAs).

We showed significant differences in the parasites’ invasion efficiency into untreated erythrocytes, which resulted in changes in the invasion profiles of some donors after treatment with either trypsin or chymotrypsin. Moreover, the levels of key erythrocyte surface receptors and their sensitivity to enzyme treatment significantly differed across donors. However, invasion efficiency did not significantly correlate with susceptibility to enzyme treatment or the expression levels of the selected erythrocyte surface receptors.

## Materials and methods

### Demographic and hematological characteristics of the study participants

The use of human erythrocytes for this study was approved by the Institutional Review Board (IRB) of the Noguchi Memorial Institute for Medical Research Ethics Committee, University of Ghana (IRB00001276) and the Ghana Health Services (GHS) ethical review committee (GHC-ERC:005/12/2017). All methods were performed in accordance with relevant guidelines and procedures as contained the approved protocol. Written informed consent was obtained from all participants. Blood samples were collected from twenty non-related asymptomatic adults, comprising fifteen males and five females, all resident in Ghana. Donors were questioned about their most recent clinically diagnosed malaria symptoms and to eliminate possible confounders, only individuals with no recent history of clinical malaria (at least two years) were considered. Additionally, erythrocytes from a single donor, used for routine parasite culturing, were included in all assays to normalize the resulting parasitemia. All but one sample were subjected to clinical diagnosis to screen for possible hemoglobin disorders while all samples were typed for ABO/Rh blood group as presented in Supplementary Table S1. In brief, the majority of the donors (14/20) presented a normal hemoglobin genotype (AA), while four donors had sickle cell trait (AS) and two other donors had an AC genotype. Blood group O^+^ was the commonest in all donors (10/20), followed by the A^+^ and B^+^ (5 and 4, respectively), while O^-^ was the least common blood group in the study participants. Of all donors, only one presented a severe deficiency of the G6PD expression. Full blood count was also performed to assess other hematological indices (Supplementary Table S1). All samples were collected in ACD vacutainers (BD Biosciences, USA) and washed three times with RPMI 1640 (Sigma Aldrich, UK) to separate the erythrocytes from the other blood components. For each sample, part of the resulting erythrocyte pellet was used for IPAs while the remaining was cryopreserved for further experiments.

### *P. falciparum* strains and culture conditions

Erythrocytes from the aforementioned donors were used to investigate the effect of blood donor variability in the invasion phenotype of both *P. falciparum* laboratory lines (3D7, Dd2 and W2mef) and clinical isolates (MISA010, MISA011 and MISA018). The clinical isolates used in this study were all collected from Accra (a low transmission setting in Ghana) between January and February 2018 and maintained in culture for 10 cycles before experiments began. All *P. falciparum* strains were maintained in culture at 4% hematocrit in complete parasite medium (RPMI 1640 containing 25 mM HEPES, 0.5% Albumax II, 2 mg/mL sodium bicarbonate and 50 µg/mL Gentamicin) and incubated at 37 °C in an atmosphere of 5.5% CO_2_, 2% O_2_ and balance N2 gas mixture. Parasites were maintained in culture using a single donor O^**+**^ erythrocytes and routinely synchronized using 5% D-Sorbitol (Sigma Aldrich, UK).

### Invasion assay set up

For each donor, erythrocytes were either untreated or treated with different enzymes, including neuraminidase (250 mU/mL), trypsin (1 mg/mL) or chymotrypsin (1 mg/mL); and labelled with 20 µM carboxyfluorescein diacetate succinimidyl ester (CFDA-SE) as described earlier^26^. For each strain, schizont stage parasites were inoculated at a 1:1 ratio into fresh enzyme-treated and labelled erythrocytes from a given donor. Experiments were conducted in triplicates in 96 well plates and repeated at least two times. Parasites were incubated for about 24 h, after which the cells were stained with 5 µM Hoechst 33342 to label the parasite’s DNA and the invasion efficiency was assessed by flow cytometry. The percentage of erythrocytes positive for both dyes was recorded as the invasion efficiency and the parasite's invasion phenotype was determined by comparing invasion rates in enzyme-treated erythrocytes to that of untreated cells. To minimize the effect of any possible confounders that may arise during the sample processing or assay set up, erythrocytes from a single donor, used for routine parasite culturing, were included in all plates and used to finally normalize the resulting parasitemia. Erythrocytes from this donor were collected at one time-point and cryopreserved in single-use vials that were thawed for subsequent assays.

### Characterization of erythrocyte surface receptors

Previously published works guided the selection of the specific erythrocyte receptors used in this study. Upon binding to the erythrocyte binding antigen EBA-175, EBL-1 and EBA140, respectively, the human glycophorins (GYP) A, B and C have previously been shown to mediate *P. falciparum* invasion through their associated sialic acid (SA) residues, hence defining the SA-dependent pathway. The complement receptor 1 (CR1), on the other hand, contributes to invasion via the SA-independent pathway through binding to the *P. falciparum* reticulocyte binding homologous 4 (PfRh4), while the *P. falciparum* invasion pathway involving the decay accelerating factor (DAF or CD55) is yet to be fully characterized. The glycophorins’ associated SA residues are susceptible to cleavage by neuraminidase whilst the receptor backbones are selectively cleaved by trypsin (GYPA, GYPC and CR1) and chymotrypsin (GYPB and CR1)^27,28^.

The surface expression of selected erythrocyte receptors was quantified by flow cytometry using specific monoclonal antibodies. Freshly washed erythrocytes were diluted to 1% hematocrit in 1X PBS containing 1% BSA and coincubated for an hour with antibodies against the human GYPAB (1:400, Sigma Aldrich UK, Catalog G7650), GYPA (Clone E4, 1:100, Santa Cruz Biotech, USA, Catalog sc-59181), GYPC (Clone E3, 1:100, Santa Cruz Biotechnology, USA, Catalog sc-59022), CR1 (Clone J3D3, 1:50, Santa Cruz Biotechnology, USA, Catalog sc-59185) or DAF (Clone NaM16-4D3, 1:50, Santa Cruz Biotechnology, USA, catalog sc-51733). The erythrocyte pellets were collected by centrifugation at 2000 rpm, washed twice with 1X PBS and subsequently coincubated with anti-mouse antibodies conjugated with either Alexa Fluor 700, APC or PE (Santa Cruz Biotechnology, USA). All incubations were done at 37 °C for an hour and protected from light exposure. The data were acquired using a BD LSR Fortessa X-20 flow cytometer (BD Biosciences, Belgium) and analyzed using FlowJo v10.5.0 (FlowJo, LLC, Ashland OR) and GraphPad Prism v.8.01 (GraphPad Software Inc., La Jolla, CA, USA).

### Antibody-dependent invasion inhibition assays

To ascertain the relative contribution of receptor density in the invasion efficiency, CFDA-labelled erythrocytes from different donors were pre-incubated at 37 °C for an hour with different concentrations of antibodies against the human GYPC, CR1 or DAF. Antibody dilutions (range 0.3125 to 5 µg/mL) were optimized to prevent formation of cell aggregates. The erythrocytes were pelleted by centrifugation at 2000 rpm for 3 min and washed with PBS prior to the addition of schizont-infected cells. The antibody-bound erythrocytes were then co-incubated at 2% hematocrit with equal volumes of parasitized erythrocytes under normal culture conditions. The parasites’ invasion rates were assessed by flow cytometry upon reinvasion.

### Statistical analyses

All statistics were performed using GraphPad Prism v.8.01 (GraphPad Software, Inc.). The data were analyzed as the mean and standard error of pooled data from at least two independent experiments conducted each in triplicates. A normality test was run on the processed data and differences in invasion efficiency into untreated or enzyme-treated erythrocytes were assessed using One-way ANOVA. Spearman correlation test was used to ascertain the relationship between variables while the Kruskal Wallis and Mann–Whitney tests were used to compare different groups. Furthermore, a multilinear regression analysis was used to predict the relative contributions of erythrocyte characteristics (blood group, Hb genotype and receptor density) to the observed variations in the invasion efficiency across donors.

## Results

### Variation in *P. falciparum* invasion efficiency across donor erythrocytes

**Figure 1:**
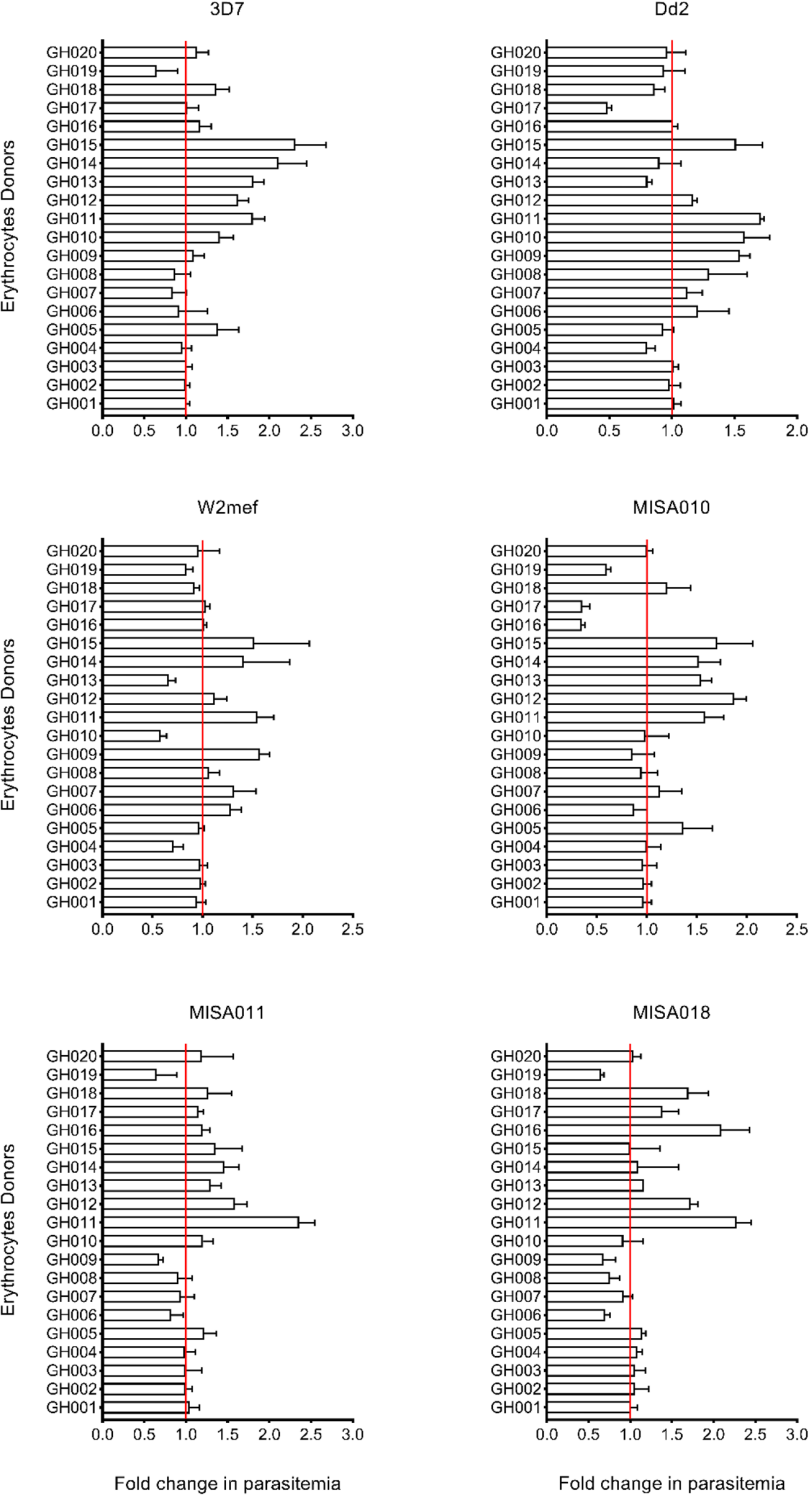
Bar graphs showing fold changes in parasitemia in untreated donor erythrocytes relative to the reference sample. Parasitemia from individual donors were normalised using a reference erythrocyte sample, which was used for routine parasite culturing and included in all assays. Fold-change in parasitemia was calculated as follow ParSMean/ParCMean, where ParSMean is the mean of uncorrected parasitemia of the sample and ParCMean the mean of parasitemia of the control sample. One-way ANOVA was used to compare the statistical analysis following normality test. Represented are summary data (mean + SD) from at least two independent experiments conducted in triplicates.

Given that erythrocytes from different donors may vary in susceptibility to invasion by a given parasite strain, we anticipated differences in invasion efficiency across donors. To enable a quantitative comparison between experiments, the resulting parasitemia for each donor was expressed relative to that of a reference donor (also used for routine parasite culturing) assayed in parallel. Untreated erythrocytes from different donors were used in these primary experiments to assess differences in *P. falciparum* invasion efficiency. For each donor, the initial parasitemia was normalized as previously described^29^. Across all strains, only few donors yielded significant increases in parasitemia as compared to the reference sample (fold change in parasitemia > 1) (Fig. 1). Moreover, all strains recorded significant differences in invasion efficiency into erythrocytes from different donors (Fig. 1; *P* < *0.0001*). Similarly, there were significant variations in invasion efficiency into erythrocytes from a given donor across the different parasite strains (Supplementary Fig S1). However, in a multivariate regressional analysis, no association was found between efficiency of invasion into untreated erythrocytes and donor blood group, Hb genotype or receptor density (Supplementary Table S2).

### Invasion phenotypes of *P. falciparum* in erythrocytes from different donors

**Figure 2:**
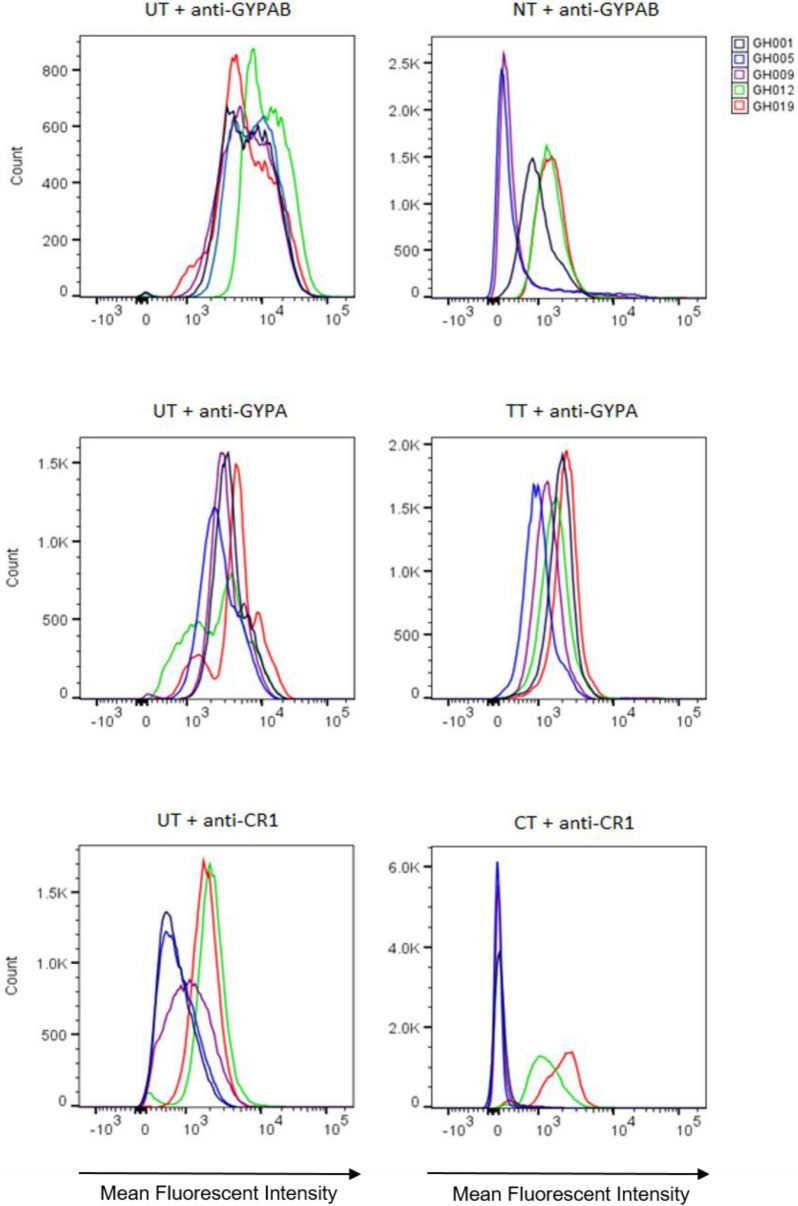
Efficacy of enzyme treatment of erythrocytes from different donors. Histogram plots showing the fluorescent intensity associated with antibodies against specific erythrocyte receptors prior to (left panel), and after (right panel) treatment with different enzymes (*NT* neuraminidase treatment, *TT* trypsin treatment, *CT* chymotrypsin treatment). Target erythrocytes from five different donors were co-incubated for an hour with mouse anti-human GYPAB (top panel), GYPA (middle panel) or CR1 (bottom panel), then washed twice and incubated with goat anti-mouse-PE conjugated antibodies. The data was collected with a BD LSR Fortessa X-20 flow cytometer and graphs plotted using FlowJo v.10.01.

**Figure 3:**
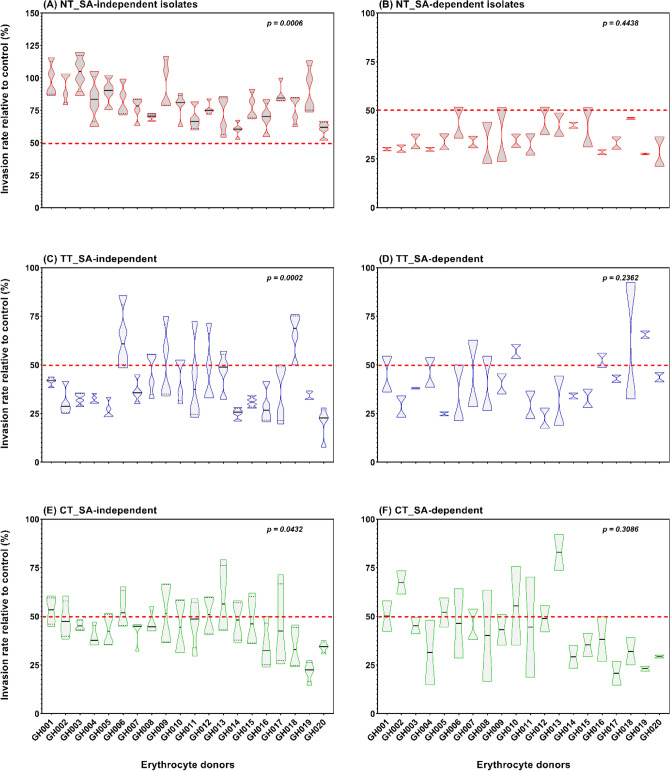
*P. falciparum* invasion phenotypes into erythrocytes from twenty different donors. The assay was set up along with a single donor erythrocyte used as a control for normalizing the resulting parasitemia. Presented are the violin plots showing the invasion efficiencies from six different *P. falciparum* strains assayed in triplicates in two independent experiments. The right panel graphs (**A**,**C**,**E**) represent the invasion phenotype of SA-independent isolates (3D7, MISA010, MISA011 and MISA018) while that of the SA-dependent isolates (Dd2 and W2mef) is represented on the left panel graphs (**B**,**D**,**F**). The red-dotted line indicates the sensitivity cut off for each given treatment. Statistical analyses were performed using One-way ANOVA. Key: *SA* sialic acid, *NT* neuraminidase treatment, *TT* trypsin treatment and *CT* chymotrypsin treatment.

To investigate the effects of blood donor variability on invasion phenotype, we determined the invasion efficiency of various *P. falciparum* strains in erythrocytes from different donors. First, we determined the sensitivities of the different donor erythrocytes to neuraminidase, which removes sialic acid (SA) residues on glycophorins, and trypsin and chymotrypsin which selectively cleave peptide backbones of other receptors^27,28^. Measurement of receptor surface expression levels showed a range of sensitivities to the three enzymes across donors (Fig. 2). We conducted assays to determine the invasion of three newly culture-adapted isolates (MISA010, MISA011 and MISA018) into enzyme-treated erythrocytes from twenty donors. Laboratory strains of *P. falciparum* (3D7, Dd2 and W2mef), with known invasion phenotypes, were also assessed. Invasion data from individual parasite strains were subsequently pooled according to their sensitivity to neuraminidase treatment (e.g. SA-dependent or SA-independent). All parasite strains tested showed differences in invasion efficiency across donors (Supplementary Tables S3-S8), and these variations remained after the data were pooled for each donor (Fig. 3).

Invasion rates into neuraminidase treated erythrocytes significantly differed between the SA-independent strains (Fig. 3A; *P* = 0.0006), while no significant difference was observed between SA-dependent parasites (Fig. 3B; *P* = 0.4438). Similarly, only SA-independent strains showed significant differences in invasion efficiency following treatment with trypsin (Fig. 3C; *P* = 0.0002) and chymotrypsin (Fig. 3E; *P* = 0.0432). Across donors, variations in invasion profiles (defined as the combination of sensitivity to the three enzymes) were mainly driven by sensitivity to trypsin and chymotrypsin treatments, since neuraminidase sensitivity remained unchanged in both individual and pooled parasite invasion rates (Supplementary Tables S3-S8).

### Relationship between receptor density and sensitivity to enzyme treatment

**Figure 4:**
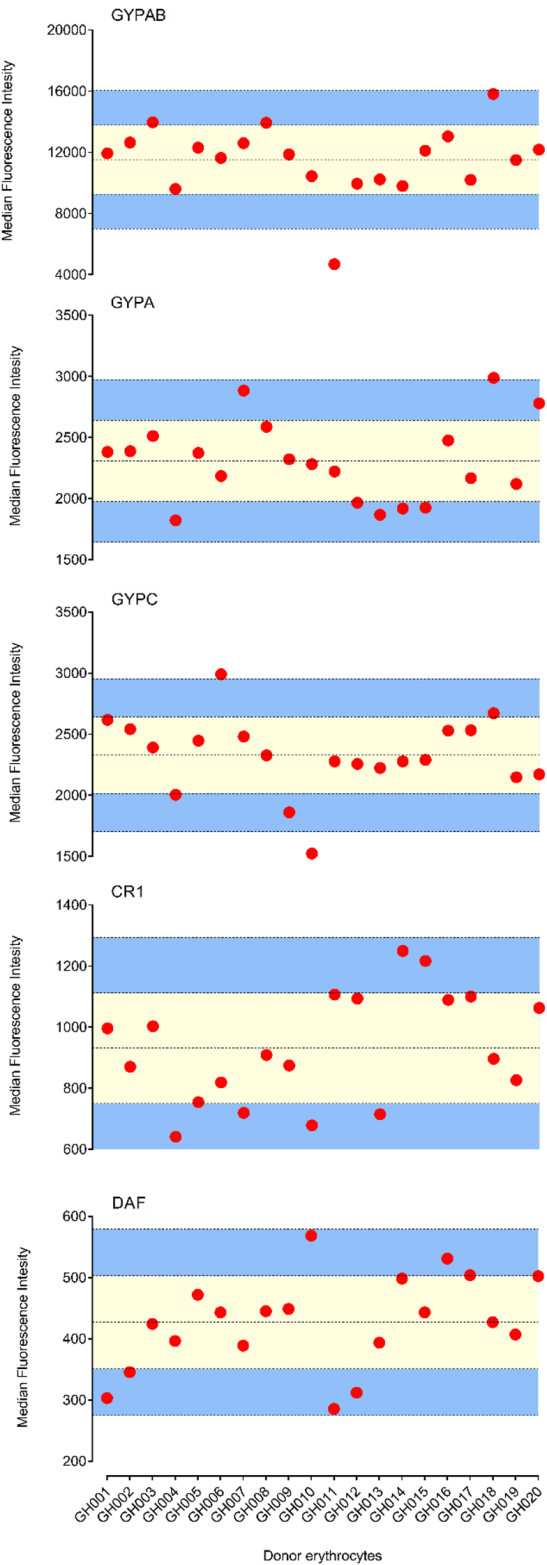
Variation of surface expression of erythrocyte receptors between donors. Dot plots of relative median fluorescent intensity (Y-axis) associated with fluorescently labelled antibodies against specific erythrocyte receptors (GYPAB, GYPA, GYPC, CR1 and DAF) from 20 blood donors (X-axis). Data were acquired by a BD LSR Fortessa X-20 flow cytometer and processed with FlowJo v.10.01. The data were stratified following normalization and donors were classified as high (Mean + 2SD, upper line), medium (between Mean ± SD, yellow area) and low expressers (Mean-2SD, bottom line). Graphs were plotted using GraphPad Prism v.8.01. Data normalization was performed as follow MFIs as follow $CorrMFIS = MFIS*\frac{MFIMean}{{MFIC}}$, where ‘‘CorrMFI’’ is the corrected mean fluorescence intensity of a given donor, ‘‘MFIS’’ is the uncorrected mean fluorescence intensity of the sample, ‘‘MFICMean’’ is the mean mean fluorescence intensity of all the control samples, and ‘‘MFIC’’ is the mean fluorescence intensity of the control for that sample.

**Figure 5:**
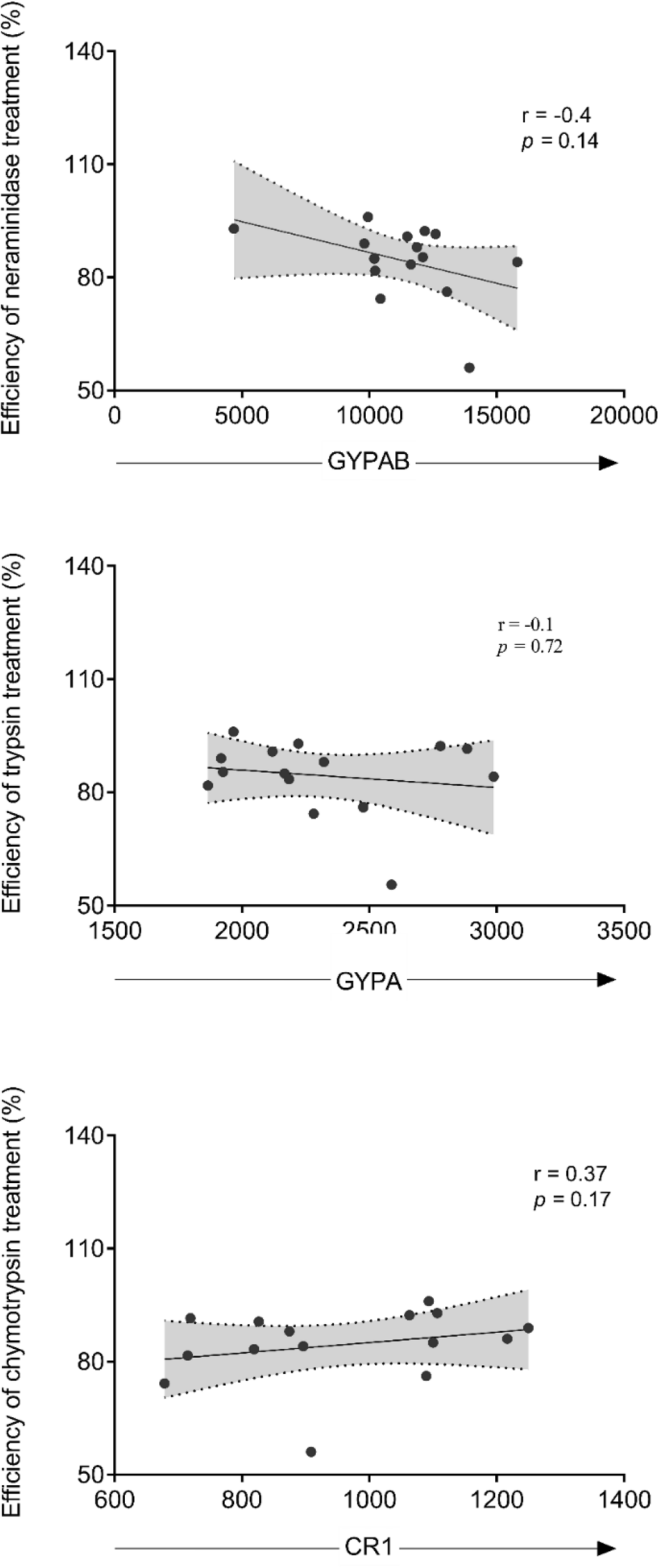
Correlation of erythrocyte receptor densities with the efficiency of enzyme treatment. The density of erythrocyte surface receptors, labelled with specific monoclonal antibodies prior to, and following enzyme treatment was quantified by flow cytometry using fluorescently labelled secondary antibodies. The efficiency of enzyme treatment was then expressed as follows: 100 – (MFI_Treated_ Erythrocytes/MFI_Control_ Erythrocytes × 100). Experiments were performed in triplicates and the resulting data were analysed with FlowJo v.10.01 and the Spearman R correlation were performed using Graph Pad Prism v.8.01.

Having shown that the sensitivity to enzyme treatment varies from donor to donor, we sought to investigate the relationship between the levels of erythrocyte surface receptors and the sensitivity to enzyme treatment. As expected, the expression levels of key erythrocyte surface receptors significantly varied between donors (Fig. 4). The median fluorescence intensity (MFI) of labelled antibodies on enzyme-treated erythrocytes was measured and expressed as the percentage of the MFI of the corresponding untreated donor erythrocytes. No significant association was found between the levels of individual receptors across donors (Supplementary Fig S2). Moreover, there was no significant association between receptor density and the efficiency of enzyme treatment (Fig. 5). Given that trypsin and chymotrypsin treatments affect the peptide backbones of the target erythrocyte receptors, we hypothesized that the observed variations in invasion profile after trypsin and chymotrypsin treatments might be driven by the differential expression of receptors on the surface of donor erythrocytes. However, no significant correlation was observed between receptor density and invasion efficiency in enzyme-treated erythrocytes (Supplementary Fig S3).

### Relationship between receptor density and *P. falciparum* invasion efficiency

**Figure 6:**
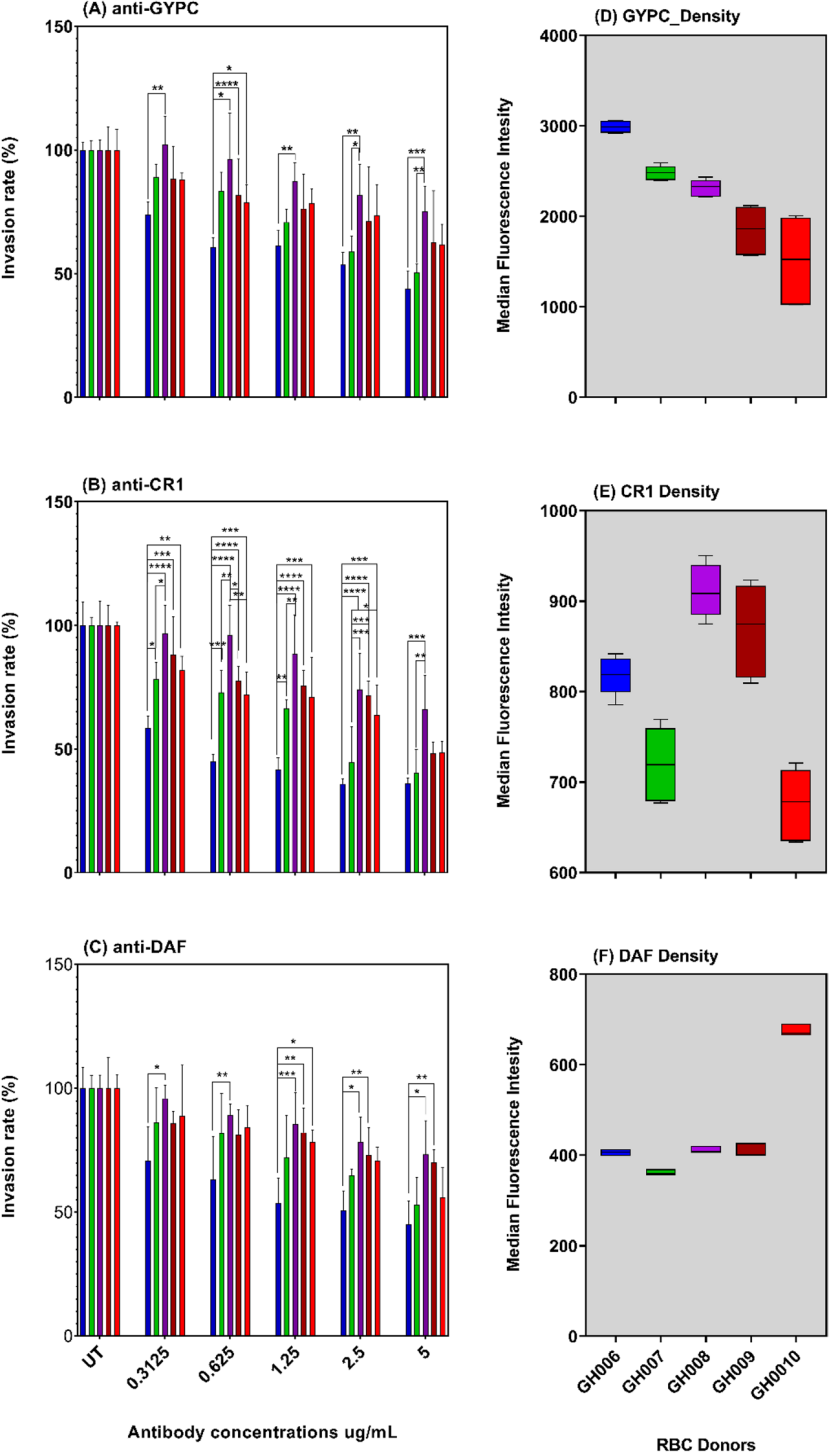
Antibody-dependent invasion inhibition assays in donor erythrocytes with different levels of surface antigens. Schizont-infected erythrocytes were co-incubated with antibody-sensitized uninfected erythrocytes from donors expressing different levels of erythrocyte receptors. The parasites’ DNA was labelled with Hoechst 33,342, 18–24 h’ post-incubation and the resulting parasitemia was quantified by flow cytometry. For each donor, the parasitemia in the corresponding mock-treated erythrocytes (similarly processed erythrocytes co-incubated with equivalent volume of RPMI 1640 with the IgY) was used to ascertain the antibody-dependent invasion inhibitory, following normalization using a single erythrocyte donor (also used for the parasites in vitro culturing). (**A**)–(**C**) represent the antibody-dependent invasion inhibition of pooled data from two different *P. falciparum* strains (3D7 and MISA011). (**D**)–(**F**) represent the differential expression of erythrocyte receptors assessed in (**A**)–(**C**).

Given the earlier reports on the relationship between the expression level of specific erythrocyte surface receptors and the invasion efficiency of *P. falciparum* strains^30–34^, we sought to rule out any effect of enzyme treatment that could mask a possible correlation between these variables. We selected five different donors (Donors 6–10, refer to Fig. 4); with different levels of expression of individual receptors and conducted antibody-mediated invasion inhibitory assays using different concentrations of specific anti-human monoclonal antibodies. As expected, there was a concentration-dependent invasion inhibition by GYPC, CR1 and DAF antibodies (Supplementary Fig S4). Overall, there were significant differences in the invasion rates across all concentrations (Fig. 6A–C). A similar pattern was observed across all antibodies, with the lowest invasion rates always recorded from donor GH006, while donor GH008 recorded the highest invasion rates (Fig. 6A–C). However, no linear relationship was observed between the invasion efficiency and the relative abundance of GYPC, CR1 or DAF erythrocyte surface receptors (Fig. 6A–F), suggesting invasion efficiency may be influenced by other donor-specific cellular properties of erythrocytes.

### Effects of ABO blood group antigens and hemoglobin genotypes on *P. falciparum* invasion phenotype

**Figure 7:**
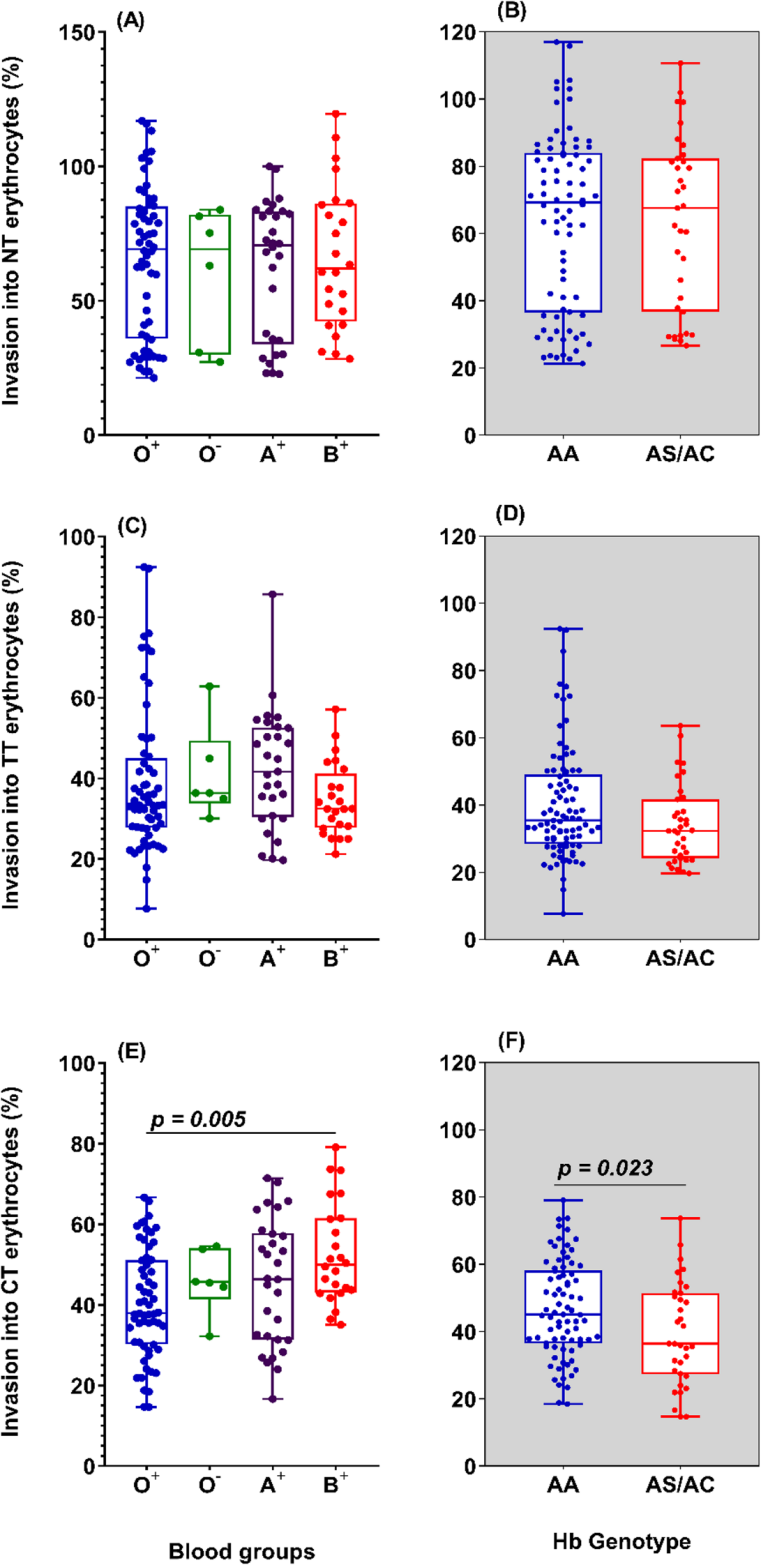
Relationship between blood group or haemoglobin genotype and invasion into enzyme-treated erythrocytes. Erythrocytes from donors of different blood groups (Left panel; O^+^  = 10, O^−^ = 1, A^+^  = 5 and B^+^  = 4) or haemoglobin genotypes (Right panel; AA = 14, AS/AC = 5) were treated with either neuraminidase (**A**,**B**), trypsin (**C**,**D**) or chymotrypsin (**E**,**F**) and co-incubated with schizont-infected *P. falciparum* strains for 18–24 h. For each set of donor erythrocytes, the invasion efficiency was assessed using flow cytometry and compared to that of mock-treated control erythrocytes. The data were analysed using Graph Pad Prism v.8.01. Differences in the invasion efficiency into erythrocytes of different blood group were assessed using the Kruskal Wallis test and where significant, pairwise comparisons were performed with the Dunn’s multiple comparison test. The Mann–Whitney test was used to compute for differences in the invasion efficiency into erythrocytes harbouring different haemoglobin genotypes.

Protection against malaria clinical manifestations has for long been associated with erythrocyte surface antigens such as those of the ABO blood group system, as well as erythrocyte hemoglobin defects^18,19,35–37^. We assessed the relative contribution of donor blood group (Fig. 7A,C,E) and hemoglobin genotype (Fig. 7B,D,F) in the observed variation in invasion phenotypes. Except for chymotrypsin treatment (*P* = 0.0053), there was no significant difference in the invasion efficiency into erythrocytes of different blood groups (Fig. 7A,C,E). This pattern was conserved when comparing invasion efficiencies into erythrocytes of different hemoglobin genotypes, where there was only significant difference following chymotrypsin treatment (*P* = 0.0231) (Fig. 7B,D,F). The multilinear regression analysis further revealed that donor blood group; hemoglobin genotype and receptor densities were not significant predictors of invasion efficiency following treatment with neuraminidase or trypsin. However, following treatment with chymotrypsin, both the blood group (|t|= 2.55, *P* = 0.02) and the receptor densities of DAF (|t|= 2.35, *P* = 0.03) significantly predicted invasion efficiency into donor erythrocytes (Supplementary table S9).

## Discussion

This study was undertaken to investigate the effect of blood donor variability in *P. falciparum* IPAs. *P. falciparum* invasion of erythrocytes is a multistep process, which includes a tightly regulated interaction between the parasite-derived ligands and specific receptors on the host cell surface. These interactions occur in a functionally redundant manner and therefore define the parasite’s invasion pathway.

Here, we present results from investigations of the effect of key erythrocyte phenotypic attributes in *P. falciparum* IPAs. Using erythrocytes from 20 unrelated donors with different blood groups and hemoglobin genotypes, we observed a wide range of variability in the invasion efficiency in both untreated and enzyme treated erythrocytes across all twenty donors. However, variation in invasion profile was only observed following treatment with either trypsin or chymotrypsin as compared to neuraminidase. This could partially be because of the mode of action of the individual enzymes. Given that trypsin and chymotrypsin are more promiscuous than neuraminidase, they could affect a wider range of receptors, including some with unknown function during the invasion. Besides, this could also be due to a small number of binding sites still present following enzyme treatment. As we found that cells from individual donors presented different levels of sensitivity to enzyme treatment, we postulated that erythrocyte receptors are differentially expressed on the surface of individual donor cells, thus driving the sensitivity to enzyme treatment. Although the expression level of erythrocyte receptors varied across donors, we observed no relationship between receptor density and sensitivity to enzyme treatment. Knockdown experiments of both basigin and CR1 have previously shown a linear relationship between receptor density and invasion efficiency into erythrocytes^30,31^. Here, there is a possibility that the absence of a relationship between receptor density and invasion efficiency was due to the effect of enzyme treatment of the erythrocytes. To rule out any effect of enzyme treatment, erythrocytes from five different donors were used in an antibody-dependent invasion inhibition assay without any prior enzyme treatment. Increasing concentrations of antibodies limits the number of receptors available for parasite invasion, therefore mimicking the effect of receptor knockdown at the individual level. In the case of a positive linear relationship between these two variables, one would expect higher invasion rates across all dilutions in individuals with a higher expression of a given receptor. Although there was an evident dose-dependent invasion inhibition by the various antibodies when donors were taken individually, there was no apparent linear relationship between receptor density and invasion efficiency. This suggests that receptor density is not the only factor influencing invasion efficiency in erythrocytes of different origin.

The relationship between *P. falciparum* and the ABO blood group system or hemoglobin genotypes remains a fascinating subject for researchers. Despite the small number of donors included in this study, we sought to investigate a possible relation between the observed invasion phenotype and the donor blood group or hemoglobin genotype. Overall, across all donors, invasion into erythrocytes of different blood groups or hemoglobin genotypes only differed significantly following chymotrypsin treatment. While majority of the hemoglobin variants conferring protection against malaria have been shown not to significantly affect erythrocyte invasion in vitro^38^, several studies have reported the parasite preference for O^+^ blood group in vitro^39,40^. More recently, different *P. falciparum* strains have been shown to differentially invade erythrocytes of the same blood group from thirty different donors^41^, suggesting that variation in invasion efficiency could be driven by other host-specific features that are yet to be defined. Furthermore, as shown by the multilinear regression analysis, the absence of relationship between invasion efficiency and the erythrocyte features tested here emphasizes that understanding the interplay between merozoite attachment and the changes in the erythrocyte biophysical properties would add complementary information about the host cell contribution to invasion. Therefore, our findings demonstrate the need to consider erythrocyte donor uniformity and anticipate challenges associated with blood donor variability in early stages of large-scale study design. While drug sensitivity assays were not investigated in this study, it is possible that variation blood donors could also influence factors such as merozoites invasion and parasite growth rate, hence, affecting drug susceptibility assays. Finally, given the heterogeneity of peripheral erythrocytes^42^, and the consequent changes in the expression of different erythrocyte surface markers^43,44^, it is of utmost importance to consider the relative contribution of the different erythrocyte sub-populations circulating in the peripheral blood as well as the differential biophysical properties on merozoite invasion.

## Supplementary Information

Supplementary Information 1.

Supplementary Information 2.
