# Supplementary material for: Blood donor variability is a modulatory factor for P. falciparum invasion phenotyping assays
Source: Sci Rep. 2021 Mar 29;11:7129. doi: 10.1038/s41598-021-86438-1 (PMC8007732; doi:10.1038/s41598-021-86438-1)
Supplement: Supplementary file 1 — Supplementary Information 1. [file 41598_2021_86438_MOESM1_ESM.pdf]

Blood donor variability is a modulatory factor for *P. falciparum* invasion phenotyping assays

Laty G. Thiam[1,2,*], Prince B. Nyarko[1,2,β], Kwadwo A. Kusi[1,3], Makhtar Niang[4], Yaw Aniweh[1,2#] and Gordon A. Awandare[1,2#]

[1]West African Centre for Cell Biology of Infectious Pathogens, College of Basic and Applied Sciences, University of Ghana, Legon

[2]Department of Biochemistry Cell and Molecular Biology, College of Basic and Applied Sciences, University of Ghana, Legon

[3]Department of Immunology, Noguchi Memorial Institute for Medical Research, University of Ghana, Legon

[4]Pôle Immunophysiopathologie et Maladies Infectieuses, Institut Pasteur de Dakar, Senegal

Running Head: Blood donor variability in *P. falciparum* invasion assays

[#]Address correspondence to Yaw Aniweh, yaniweh@ug.edu.gh

[#]Address correspondence to Gordon A. Awandare, gawandare@ug.edu.gh

[*]Present address: G4 MEGA Vaccines, Institut Pasteur de Dakar, Senegal

[β]Present address: Laboratory of Pathogen-Host Interaction, UMR5235, CNRS, University of Montpellier

Table S1: Clinical and hematological indices of study participants

ID	Gender	Blood group	Sickling	Hb genotype	G6PD	WBC (10^9/L)	RBC (10^12/L)	HGB (g/dL)	HCT (%)	MCV (fL)	MCH (pg)	MCHC (g/dL)	PLT (10^9/L)
**GH000****	M	O+	-	AA	Normal	4.34	5.16	13.0	42.8	83.0	25.2	30.4	153
**GH001**	F	O+	-	AA	Normal	7.98	3.93	11.5	38.9	98.9	29.3	29.6	342
**GH002**	M	B+	-	AA	Normal	5.91	6.04	15.0	51.5	85.2	24.8	29.1	231
**GH003**	M	B+	+	AS	Normal	3.16	5.34	15.1	46.7	87.4	28.3	32.3	220
**GH004**	M	O+	+	AS	Normal	4.38	6.26	13.7	44.8	71.5	21.9	30.6	218
**GH005**	M	O+	-	AA	Normal	6.82	5.31	14.3	47.1	88.7	26.9	30.4	202
**GH006**	F	A+	-	AA	Normal	6.88	3.83	12.3	39.4	102.8	32.1	31.2	273
**GH007**	M	O-	-	AA	Normal	5,07	4,77	14,5	43,6	91,4	30,4	33,3	228
**GH008**	M	A+	-	AA	NA	NA	NA	NA	NA	NA	NA	NA	NA
**GH009**	M	O+	-	AA	Normal	5,59	3,76	13,3	39,9	106,2	35,4	33,3	317
**GH010**	M	A+	+	AS	Normal	5.57	6.30	16.3	51.7	82.0	25.9	31.5	258
**GH011**	M	O+	-	AA	Normal	3.86	5.21	15.4	48.3	92.7	29.6	31.9	169
**GH012**	M	O+	-	AA	Normal	4.74	5.94	15.2	49.5	83.4	25.6	30.7	197
**GH013**	F	B+	-	AA	Normal	5.53	4.57	13.5	43.0	94.0	29.5	31.4	244
**GH014**	F	B+	+	AS	Normal	5.73	4.61	12.9	40.2	87.1	28.0	32.1	251
**GH015**	M	O+	-	AA	Normal	7.90	4.95	14.6	46.8	94.6	29.5	31.2	253
**GH016**	M	A+	-	AC	Normal	5.49	4.74	13.2	39.6	83.5	27.8	33.3	244
**GH017**	M	A+	-	AA	Full Defect	3,02	3,85	9,9	31,5	81,8	25,7	31,4	209
**GH018**	F	O+	NA	NA	NA	NA	NA	NA	NA	NA	NA	NA	NA
**GH019**	M	O+	-	AC	Normal	7.70	5.48	14.6	45.2	82.5	26.6	32.3	260
**GH020**	M	O+	-	AA	Normal	5.94	5.35	15.7	49.1	91.8	29.3	32.0	187

** Erythrocytes from this donor were used for routine parasite culturing and for data normalization

Table S2: Summary outputs from multilinear regression analysis showing the relationship between invasion efficiency into untreated erythrocytes and blood group, Hb genotype o receptor density. The data were computed using least square regression method to predict the variation of the invasion rate relative to the independent variables.

UT_3D7: R2 = 0.49, Adjusted R2 = 0.20, F (7, 12) = 1.67, P value = 0.21

Variable	Estimate (β)	Standard error	95% confidence interval	|t|	P value
**Blood group**	-0,05132	0,1171	-0,3064 - 0,2038	0,4383	0,6689
**Hb Genotype**	-0,2661	0,2345	-0,7771 - 0,2449	1,135	0,2787
**GYPAB**	-1,721e-005	6,056e-005	-0,0001492 - 0,0001147	0,2841	0,7812
**GYPA**	-0,0004831	0,0003743	-0,001299 - 0,0003324	1,291	0,2211
**GYPC**	-0,0002623	0,0004069	-0,001149 - 0,0006241	0,6448	0,5312
**CR1**	0,001089	0,0005766	-0,0001668 - 0,002346	1,889	0,0832
**DAF**	0,001071	0,001679	-0,002587 - 0,004728	0,6378	0,5356

UT_Dd2: R2 = 0.33, Adjusted R2 = -0.67, F (7, 12) = 0.8291, P value = 0.58

Variable	Estimate (β)	Standard error	95% confidence interval	|t|	P value
**Blood group**	0.05958	0.09235	-0.1416 - 0.2608	0.6451	0.5310
**Hb Genotype**	-0.1441	0.1850	-0.5472 - 0.2590	0.7787	0.4512
**GYPAB**	-1.736e-005	4.777e-005	-0.0001214 - 8.673e-005	0.3634	0.7226
**GYPA**	0.0001856	0.0002952	-0.0004577 - 0.0008288	0.6286	0.5414
**GYPC**	-0.0006127	0.0003209	-0.001312 - 8.660e-005	1.909	0.0805
**CR1**	0.0003809	0.0004548	-0.0006101 - 0.001372	0.8374	0.4187
**DAF**	-0.001004	0.001324	-0.003890 - 0.001881	0.7585	0.4628

UT_W2mef: R2 = 0.45, Adjusted R2 = 1.13, F (7, 12) = 1.392, P value = 0.29

Variable	Estimate (β)	Standard error	95% confidence interval	|t|	P value
**Blood group**	0,04067	0,07859	-0,1306 - 0,2119	0,5175	0,6142
**Hb Genotype**	-0,1877	0,1575	-0,5308 - 0,1553	1,192	0,2562
**GYPAB**	-2,169e-005	4,066e-005	-0,0001103 - 6,689e-005	0,5335	0,6035
**GYPA**	5,069e-005	0,0002513	-0,0004967 - 0,0005981	0,2018	0,8435
**GYPC**	-4,383e-005	0,0002731	-0,0006389 - 0,0005513	0,1605	0,8752
**CR1**	0,0008825	0,0003871	3,914e-005 - 0,001726	2,280	0,0417
**DAF**	-0,0003185	0,001127	-0,002774 - 0,002137	0,2826	0,7823

UT_MISA010: R2 = 0.2, Adjusted R2 = 0.04, F (7, 12) = 1.127, P value = 0.41

Variable	Estimate (β)	Standard error	95% confidence interval	|t|	P value
**Blood group**	-0,04625	0,1176	-0,3025 - 0,2100	0,3932	0,7010
**Hb Genotype**	-0,2919	0,2356	-0,8053 - 0,2215	1,239	0,2392
**GYPAB**	7,748e-006	6,084e-005	-0,0001248 - 0,0001403	0,1273	0,9008
**GYPA**	-0,0003037	0,0003760	-0,001123 - 0,0005155	0,8078	0,4350
**GYPC**	-0,0003782	0,0004088	-0,001269 - 0,0005125	0,9251	0,3731
**CR1**	0,0004047	0,0005793	-0,0008574 - 0,001667	0,6987	0,4981
**DAF**	-0,001651	0,001687	-0,005326 - 0,002024	0,9790	0,3469

UT_MISA011: R2 = 0.65, Adjusted R2 = 0.45, F (7, 12) = 3.186, P value = 0.04

Variable	Estimate (β)	Standard error	95% confidence interval	|t|	P value
**Blood group**	-0,04720	0,08016	-0,2218 - 0,1275	0,5888	0,5669
**Hb Genotype**	-0,03375	0,1606	-0,3836 - 0,3161	0,2101	0,8371
**GYPAB**	-0,0001302	4,147e-005	-0,0002206 - -3,989e-005	3,141	0,0085
**GYPA**	0,0003764	0,0002562	-0,0001819 - 0,0009347	1,469	0,1676
**GYPC**	6,202e-005	0,0002786	-0,0005449 - 0,0006690	0,2226	0,8276
**CR1**	0,0007183	0,0003948	-0,0001418 - 0,001578	1,820	0,0939
**DAF**	-2,505e-005	0,001149	-0,002529 - 0,002479	0,02180	0,9830

UT_MISA018: R2 = 0.43, Adjusted R2 = 0.09, F (7, 12) = 1.290, P value = 0.33

Variable	Estimate	Standard error	95% confidence interval	|t|	P value
**Blood group**	-0,06779	0,1246	-0,3392 - 0,2036	0,4079	0,6905
**Hb Genotype**	0,1987	0,2495	-0,3450 - 0,7424	0,5442	0,5963
**GYPAB**	-0,0001291	6,443e-005	-0,0002695 - 1,133e-005	0,7961	0,4414
**GYPA**	0,0006449	0,0003982	-0,0002227 - 0,001513	2,003	0,0683
**GYPC**	0,0004201	0,0004329	-0,0005231 - 0,001363	1,620	0,1313
**CR1**	0,0007125	0,0006134	-0,0006240 - 0,002049	0,9704	0,3510
**DAF**	-0,0001109	0,001786	-0,004002 - 0,003781	1,162	0,2680

Table S3-S8: Summary of invasion profiles of individual *P. falciparum* strains into enzyme-treated erythrocyte from different donors.

Data represent mean values ± the standard deviation from two independent experiments conducted in triplicates. A cut-off of 50% was taken as a threshold to classify the observed profile as sensitive (≤50%) or resistance (>50%) for each given treatment.

Table S3: MISA010

			% invasion relative to untreated control		
**Donor Erythrocyte**	**Gender**	**Invasion profile**	**Neuraminidase (250 mU/ml)**	**Trypsin (1mg/ml)**	**Chymotrypsin (1mg/ml)**
**O+/AA**	F	NrTsCr	103.1 ± 16.3	41.7 ± 4.1	60.4 ± 17.0
**B+/AA**	M	NrTsCr	75.0 ± 8.1	25.0 ± 7.0	61.3 ± 11.2
**B+AS**	M	NrTsCs	119.6 ± 22.4	28.6 ± 4.2	42.9 ± 8.6
**O+AS**	M	NrTsCs	62.5 ± 6.1	35.4 ± 15.6	35.4 ± 18.9
**O+AA**	M	NrTsCr	75.7 ± 23.3	23.6 ± 4.3	51.7 ± 19.5
**A+/AA**	F	NrTrCr	71.43 ± 18.31	85.71 ± 1,23	57.14 ± 17.73
**O-/AA**	M	NrTsCs	75.22 ± 9.87	44.95 ± 1.47	45.45 ± 12.55
**A+/AA**	M	NrTrCs	66.66 ± 17.36	55.55 ± 4.78	46.29 ± 4,78
**O+/AA**	M	NrTrCr	80.55 ± 13.58	58.33 ± 29.48	65.74 ± 14.3
**A+/AS**	M	NrTrCr	81.31 ± 5.62	52.52 ± 16.52	57.57 ± 16.26
**O+/AA**	M	NrTrCr	81.62 ± 5,87	72.46 ± 14.54	51.0 ± 5.85
**O+/AA**	M	NrTrCs	75.12 ± 2.17	71.52 ± 13.18	40.59 ± 7.25
**B+/AA**	M	NrTsCr	54.35 ± 4.24	47.06 ± 8.5	67.64 ± 7.15
**B+/AS**	F	NrTsCs	60.71 ± 0.92	25 ±13.04	41.67 ± 16.34
**O+/AA**	M	NrTsCs	71.23 ± 21.05	32 ± 15.31	36 ± 7.37
**A+/AC**	M	NrTsCr	83.33 ± 11.72	41.67 ± 13.44	50.42 ± 13.1
**A+/AA**	M	NrTrCr	83.33 ± 14.91	50.34 ± 19.72	52.5 ± 5.1
**O+/AA**	F	NrTrCs	85.36 ± 16.71	65.13 ± 12.19	45.65 ± 5.59
**O+/AC**	M	NrTsCs	79.48 ± 11.14	35.90 ± 14.56	21.88 ± 9.97
**O+/AA**	M	NrTsCs	63.49 ± 15.69	22.22 ± 0.65	34.75 ± 7.70

Table S4: MISA011

		% invasion relative to untreated control			
**Donor erythrocytes**	Gender	Invasion profile	Neuraminidase (250 mU/ml)	Trypsin (1mg/ml)	Chymotrypsin (1mg/ml)
**O+/AA**	F	NrTsCr	115.89 ± 2.33	42.39 ± 10.26	56.75 ± 26.27
**B+/AA**	M	NrTsCr	103.03 ± 8.11	25.06 ± 9.42	50.35 ± 6.36
**B+AS**	M	NrTsCs	110.71 ± 24.1	35.71 ± 19.19	46.43 ± 17.56
**O+AS**	M	NrTsCs	105.14 ± 8.52	30.28 ± 8.39	40 ± 14.97
**O+AA**	M	NrTsCs	101.96 ± 18.13	33.44 ± 5.92	35.89 ± 9.95
**A+/AA**	F	NrTrCs	99.06 ± 14.35	55.14 ± 15.69	46.48 ±7.23
**O-/AA**	M	NrTsCs	83.89 ± 14.81	30.09 ± 11.83	32.2 ± 13.89
**A+/AA**	M	NrTsCs	72.55 ± 11.24	32.84 ± 8.9	42.53 ± 6.85
**O+/AA**	M	NrTsCs	105.60 ± 9.64	37.58 ± 9.36	37.36 ± 9.78
**A+/AS**	M	NrTsCs	88.04 ± 10.32	36.14 ±21.78	31.26 ± 11.93
**O+/AA**	M	NrTsCs	59.76 ± 6.28	23.15 ± 6.24	29.67 ± 5.31
**O+/AA**	M	NrTsCs	71.73 ± 9.29	33.30 ± 11.85	43.01 ± 16.62
**B+/AA**	M	NrTrCs	81.81 ± 3.84	50.65 ± 9.00	79.11 ± 7.65
**B+/AS**	F	NrTsCs	60.53 ± 3.84	26.29 ± 9.55	36.45 ± 13.45
**O+/AA**	M	NrTrCs	91.38 ± 16.01	29.79 ± 14.67	37.70 ± 11.05
**A+/AC**	M	NrTsCs	72.56 ± 3.98	20.76 ± 2.02	28.31 ± 1.70
**A+/AA**	M	NrTsCs	82.31 ± 7.00	19.72 ± 3.85	32.60 ± 4.98
**O+/AA**	F	NrTrCs	83.20 ± 13.44	72.56 ± 7.87	26.05 ± 2.31
**O+/AC**	M	NrTsCs	113.26 ±12.97	32.33 ± 19.87	27.45 ± 12.71
**O+/AA**	M	NrTsCs	66.80 ± 3.25	23.33 ± 7.43	34.31 ± 11.03

Table S5: MISA018

		% invasion relative to untreated control			
**Donor Erythrocyte**	**Gender**	**Invasion profile**	**Neuraminidase (250 mU/ml)**	**Trypsin (1mg/ml)**	**Chymotrypsin (1mg/ml)**
**O+/AA**	F	NrTsCr	90.48 ± 7.84	38.28 ± 5.14	44.8 ± 5.13
**B+/AA**	M	NrTsCr	87.47 ± 11.81	27.58 ± 12.07	38.24 ± 12.42
**B+AS**	M	NrTsCs	99.08 ± 6.46	34.31 ± 14.28	43.72 ± 11.36
**O+AS**	M	NrTsCs	78.91 ± 4.89	33.15 ± 13.31	35.51 ± 6.58
**O+AA**	M	NrTsCr	92.89 ± 4.59	27.46 ± 6.22	35,45 ± 5.68
**A+/AA**	F	NrTrCr	75.59 ± 4.59	66.67 ± 7.07	65.38 ± 16.79
**O-/AA**	M	NrTsCs	63.09 ± 11.75	36.36 ± 8.76	45.80 ± 9.87
**A+/AA**	M	NrTrCr	71.21 ± 18.51	53.98 ± 16.55	55.21 ± 9.61
**O+/AA**	M	NrTrCr	116.98 ± 24.10	75.26 ± 30.78	66.67 ± 9,24
**A+/AS**	M	NrTrCr	81.34 ± 25.29	52.76 ± 26.97	58.5 ± 13.99
**O+/AA**	M	NrTsCs	64.61 ± 7.60	46.15 ± 2.65	43.25 ± 10.92
**O+/AA**	M	NrTrCr	84.31 ± 14.20	50.15 ± 15.49	60.74 ± 8.94
**B+/AA**	M	NrTrCs	85.71 ± 12.65	57.14 ± 4.92	42.86 ± 5,53
**B+/AS**	F	NrTsCr	52.57 ± 10.63	21.2 ± 2.99	54.55 ± 12.96
**O+/AA**	M	NrTsCr	68.36 ± 27.85	34.48 ± 8,51	62.07 ± 16.79
**A+/AC**	M	NrTsCs	54.54 ± 5,63	20.09 ± 4,30	36.36 ± 14.76
**A+/AA**	M	NrTsCr	85.71 ± 2.95	45.71 ± 6.76	71.42 ± 17.21
**O+/AA**	F	NrTrCs	62.50 ± 15.86	50.36 ± 16.45	40.93 ± 23.05
**O+/AC**	M	NrTsCs	73.85 ± 10.27	32.31 ± 11.19	23.08 ± 7.96
**O+/AA**	M	NrTsCs	60.29 ± 5.31	27.94 ± 9.33	37.65 ± 10.11

Table S6: 3D7

			% invasion relative to untreated control		
**Donor Erythrocyte**	**Gender**	**Invasion profile**	**Neuraminidase (250 mU/ml)**	**Trypsin (1mg/ml)**	**Chymotrypsin (1mg/ml)**
**O+/AA**	F	NrTsCs	86.54 ± 9.82	43.76 ± 11.41	49.9 ± 7.22
**B+/AA**	M	NrTsCs	79.24 ± 16.54	30.05 ± 9.74	44.26 ± 4.43
**B+AS**	M	NrTsCs	86.36 ± 11.64	31.76 ± 8.91	48.37 ±7.05
**O+AS**	M	NrTsCs	88.02 ± 15.45	30.57 ± 2.00	47.19 ± 4.61
**O+AA**	M	NrTsCs	88.07 ± 14.44	23.25 ± 11.51	48.70 ± 7.05
**A+/AA**	F	NrTsCs	86.93 ± 8.26	48.52 ± 7.97	45 ± 8.21
**O-/AA**	M	NrTsCs	81.39 ± 8.92	35 ± 8.21	44.44 ± 6.34
**A+/AA**	M	NrTsCs	69.98 ± 6.48	40.98 ± 5.75	43.06 ± 3.06
**O+/AA**	M	NrTsCs	78.67 ± 6.69	34.17 ± 6.44	36.61 ± 7.87
**A+/AS**	M	NrTsCs	62.36 ± 9.78	30.00 ± 6.27	31.37 ± 5.95
**O+/AA**	M	NrTsCr	68.61 ± 8.55	28.72 ± 9.3	59.18 ± 7.89
**O+/AA**	M	NrTsCr	74.68 ± 9.55	33.03 ± 6.81	58.81 ± 6.34
**B+/AA**	M	NrTsCs	63.43 ± 43	32.37 ± 6.19	45.09 ± 8.43
**B+/AS**	F	NrTsCr	67.57 ± 10.84	28.14 ± 21.94	57.95 ± 18.05
**O+/AA**	M	NrTsCr	82.13 ± 11.59	27.8 ± 9.09	54.52 ± 13.83
**A+/AC**	M	NrTsCs	68.17 ± 3.87	24.18 ± 2.97	24.01 ± 2.04
**A+/AA**	M	NrTsCs	100 ± 6.70	26.31 ± 0.39	25.66 ± 4.72
**O+/AA**	F	NrTsCs	69.89 ± 10.98	76 ± 15.4	24.1 ± 1.04
**O+/AC**	M	NrTsCs	99.23 ± 3.79	36.71 ± 4.09	14.68 ± 7.78
**O+/AA**	M	NrTsCs	51.87 ± 14.31	7.67 ± 3.21	30.70 ±2.69

Table S7: Dd2

			% invasion relative to untreated control		
**Donor Erythrocyte**	**Gender**	**Invasion profile**	**Neuraminidase (250 mU/ml)**	**Trypsin (1mg/ml)**	**Chymotrypsin (1mg/ml)**
**O+/AA**	F	NsTsCs	30.94 ± 4.34	32.25 ± 1.85	37.87 ± 4.47
**B+/AA**	M	NsTsCr	31.03 ± 3.08	37.93 ± 16.82	51.72 ± 2.05
**B+AS**	M	NsTsCr	36.71 ± 13.75	44.05 ± 5.69	51.39 ± 2.05
**O+AS**	M	NsTsCr	31.03 ± 3.08	37.93 ± 6.04	51.72 ± 2.05
**O+AA**	M	NsTsCr	36.71 ± 5.76	44.05 ± 5.69	51.4 ± 6.69
**A+/AA**	F	NsTsCs	23.08 ± 6.89	30.77 ± 6.95	38.46 ± 7.94
**O-/AA**	M	NsTsCr	27.27 ± 11,56	36.06 ± 8.21	54.26 ± 4.69
**A+/AA**	M	NsTsCr	23.07 ± 13.24	38.46 ± 7.94	53.85 ± 7.94
**O+/AA**	M	NsTsCs	23.80 ± 3.25	21.42 ± 5.61	28.57 ± 7.38
**A+/AS**	M	NsTsCr	26.67 ± 8.27	35.53 ± 6.49	53.33 ± 5.96
**O+/AA**	M	NsTsCs	36.65 ± 2.82	17.79 ± 10.54	18.43 ± 5.31
**O+/AA**	M	NsTsCr	35.57 ± 1.50	28.07 ± 3.51	56.14 ± 13.54
**B+/AA**	M	NsTsCr	41.03 ± 4.13	32.5 ± 3.09	67.5 ± 18.43
**B+/AS**	F	NsTsCr	46.15 ± 2.16	42.31 ± 15,75	61.54 ± 10.14
**O+/AA**	M	NsTsCr	41.03 ± 5.05	14.80 ± 6.39	59.59 ±10.87
**A+/AC**	M	NsTsCs	28.57 ± 2.83	38.09 ± 12.13	16.67 ± 2.56
**A+/AA**	M	NsTsCr	35.18 ± 11.04	35.12 ± 12.59	70.37 ± 13.18
**O+/AA**	F	NsTrCs	42.10 ± 4.24	92.10 ± 4.08	23.39 ± 3.51
**O+/AC**	M	NsTsCs	29.30 ± 4.96	49.82 ± 11.23	14.65 ± 4.34
**O+/AA**	M	NsTsCs	25.05 ± 11.88	24.46 ± 4.59	28.59 ± 8.27

Table S8: W2mef

			% invasion relative to untreated control		
**Donor Erythrocyte**	**Gender**	**Invasion profile**	**Neuraminidase (250 mU/ml)**	**Trypsin (1mg/ml)**	**Chymotrypsin (1mg/ml)**
**O+/AA**	F	NsTsCr	29.14 ± 3.37	36.05 ± 11.15	58.02 ± 14.32
**B+/AA**	M	NsTsCr	28.36 ± 6.49	34.14 ± 9.6	73.41 ± 10.43
**B+AS**	M	NsTsCs	30.16 ± 5.17	37.67 ± 8.84	49.53 ± 12.87
**O+AS**	M	NsTsCs	28.89 ± 3.61	38.52 ± 0	48.15 ± 3.1
**O+AA**	M	NsTsCs	29.63 ± 3.13	25.92 ± 3.89	44.44 ± 2.88
**A+/AA**	F	NsTrCr	35.71 ± 8.21	50.32 ± 3,69	64.28 ± 13.29
**O-/AA**	M	NsTrCr	30.77 ± 9.54	62.82 ± 14.15	53.85 ± 4.42
**A+/AA**	M	NsTrCr	22.72 ± 4.06	54.54 ± 0.78	63.64 ± 17.84
**O+/AA**	M	NsTsCr	23.67 ± 6.49	45.45 ± 7,33	51.14 ± 10.58
**A+/AS**	M	NsTrCr	37.87 ± 3.91	60.61 ± 11.37	65.75 ± 22.12
**O+/AA**	M	NsTsCs	27.08 ± 13.13	22.32 ± 12.01	18.75 ± 17.40
**O+/AA**	M	NsTsCr	37.44 ± 10.42	27.78 ± 2.14	55.56 ± 17.46
**B+/AA**	M	NsTsCr	48.82 ± 7.63	44.44 ± 7.63	73.73 ± 18.10
**B+/AS**	F	NsTsCs	40.79 ± 14.31	32.46 ± 2.72	35.05 ± 16.70
**O+/AA**	M	NsTsCs	31.25 ± 17.39	37.5 ± 14.82	41.44 ± 15.00
**A+/AC**	M	NsTsCs	29.83 ± 11.68	48.72 ± 8.18	26.76 ± 4.03
**A+/AA**	M	NsTsCs	30.08 ± 9.89	44.84 ± 3.37	26.89 ± 3.55
**O+/AA**	F	NsTrCs	46.38 ± 3.56	92.44 ± 7.85	37.61 ± 4.31
**O+/AC**	M	NsTrCs	28.07 ± 4.01	63.64 ± 8.73	21.832 ± 5.38
**O+/AA**	M	NsTsCs	21.33 ± 0.49	41.33 ± 8.39	30.11 ± 4.87

Key: N: neuraminidase, T: trypsin, C: chymotrypsin, s: sensitive, r: resistantData represent mean values ± the standard deviation from two independent experiments conducted in triplicates. A cut-off of 50% was taken as a threshold to classify the observed profile as sensitive (≤50%) or resistance (>50%) for each given treatment.

Table S9: Summary outputs from multilinear regression analysis. The data were computed using least square regression method to predict the variation of the invasion rate relative to the independent variables.

Neuraminidase treatment: R^2 = 0.21, Adjusted R^2 = -0.24, F (7, 12) = 0.46, P value = 0.84

Variable	Estimate (β)	Standard error	95% confidence interval	|t|	P value
**Invasion rate**	85.99	37.09	5.176 - 166.8	2.318	0.0389
**Blood group**	0.2004	3.391	-7.187 - 7.588	0.05910	0.9538
**Hb Genotype**	1.704	6.793	-13.10 - 16.50	0.2508	0.8062
**GYPAB**	0.002168	0.001754	-0.001653 - 0.005990	1.236	0.2400
**GYPA**	-0.006624	0.01084	-0.03024 - 0.01699	0.6112	0.5525
**GYPC**	0.0007943	0.01178	-0.02488 - 0.02647	0.06741	0.9474
**CR1**	-0.006623	0.01670	-0.04300 - 0.02976	0.3966	0.6986
**DAF**	-0.05103	0.04862	-0.1570 - 0.05490	1.050	0.3146

Trypsin treatment: R^2 = 0.53, Adjusted R^2 = 0.25, F (7, 12) = 1.93, P value = 0.15

Variable	Estimate	SE	95% confidence interval	|t|	P value
**Invasion rate**	40.01	31.31	-28.20 - 108.2	1.278	0.2254
**Blood group**	0.2279	2.862	-6.007 - 6.463	0.07965	0.9378
**Hb Genotype**	-0.2142	5.733	-12.71 - 12.28	0.03737	0.9708
**GYPAB**	0.0007473	0.001480	-0.002478 - 0.003973	0.5048	0.6228
**GYPA**	0.002327	0.009148	-0.01761 - 0.02226	0.2544	0.8035
**GYPC**	0.001903	0.009945	-0.01977 - 0.02357	0.1913	0.8515
**CR1**	-0.01686	0.01409	-0.04757 - 0.01385	1.196	0.2546
**DAF**	-0.01154	0.04103	-0.1009 - 0.07786	0.2813	0.7833

Chymotrypsin treatment: R^2 = 0.21, Adjusted R^2 = -0.24, F (7, 12) = 0.46, P value = 0.84

Variable	Estimate	SE	95% confidence interval	|t|	P value
**Invasion rate**	70.73	25.62	14.91 - 126.6	2.761	0.0173
**Blood group**	5.976	2.342	0.8732 - 11.08	2.552	0.0254
**Hb Genotype**	7.311	4.692	-2.912 - 17.53	1.558	0.1452
**GYPAB**	0.0008028	0.001211	-0.001837 - 0.003442	0.6627	0.5201
**GYPA**	-0.01026	0.007487	-0.02657 - 0.006056	1.370	0.1958
**GYPC**	-0.003504	0.008139	-0.02124 - 0.01423	0.4306	0.6744
**CR1**	0.009594	0.01153	-0.01554 - 0.03472	0.8318	0.4217
**DAF**	-0.08046	0.03358	-0.1536 - -0.007294	2.396	0.0338
