# Supplementary material for: Blood donor variability is a modulatory factor for P. falciparum invasion phenotyping assays
Source: Sci Rep. 2021 Mar 29;11:7129. doi: 10.1038/s41598-021-86438-1 (PMC8007732; doi:10.1038/s41598-021-86438-1)
Supplement: Supplementary file 2 — Supplementary Information 2. [file 41598_2021_86438_MOESM2_ESM.pdf]

Blood donor variability is a modulatory factor for *P. falciparum* invasion phenotyping assays

Laty G. Thiam[1,2,*], Prince B. Nyarko[1,2,β], Kwadwo A. Kusi[1,3], Makhtar Niang[4], Yaw Aniweh[1,2#] and Gordon A. Awandare[1,2#]

[1]West African Centre for Cell Biology of Infectious Pathogens, College of Basic and Applied Sciences, University of Ghana, Legon

[2]Department of Biochemistry Cell and Molecular Biology, College of Basic and Applied Sciences, University of Ghana, Legon

[3]Department of Immunology, Noguchi Memorial Institute for Medical Research, University of Ghana, Legon

[4]Pôle Immunophysiopathologie et Maladies Infectieuses, Institut Pasteur de Dakar, Senegal

Running Head: Blood donor variability in *P. falciparum* invasion assays

[#]Address correspondence to Yaw Aniweh, yaniweh@ug.edu.gh

[#]Address correspondence to Gordon A. Awandare, gawandare@ug.edu.gh

[*]Present address: G4 MEGA Vaccines, Institut Pasteur de Dakar, Senegal

[β]Present address: Laboratory of Pathogen-Host Interaction, UMR5235, CNRS, University of Montpellier

Supplementary figures

Figure S1

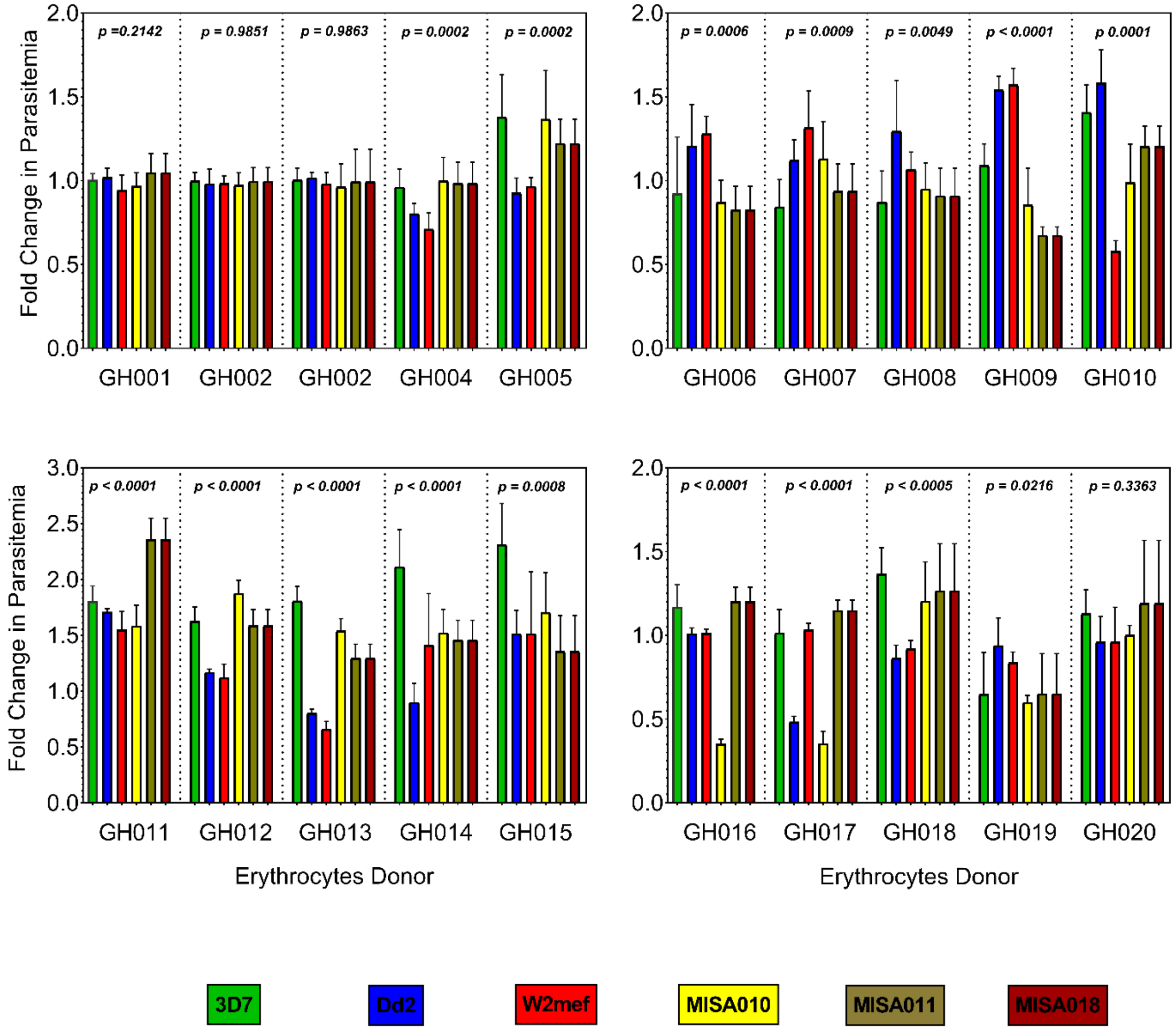

Figure S1: Bar graphs showing fold changes in the parasites' invasion rates into individual donor's erythrocytes relative to the reference sample. Parasitemia from individual donors were normalised using a reference erythrocyte sample, also used for routine parasite culturing and included in all assays. Fold-change in parasitemia was calculated as follow ParSMean/ParCMean, where ParSMean is the mean of uncorrected parasitemia of the sample and ParCMean the mean of parasitemia of the control sample. One-way ANOVA was used to compare the statistical analysis following normality test. Represented are summary data (mean + SD) from at least two independent experiments conducted in triplicates.

Figure S2

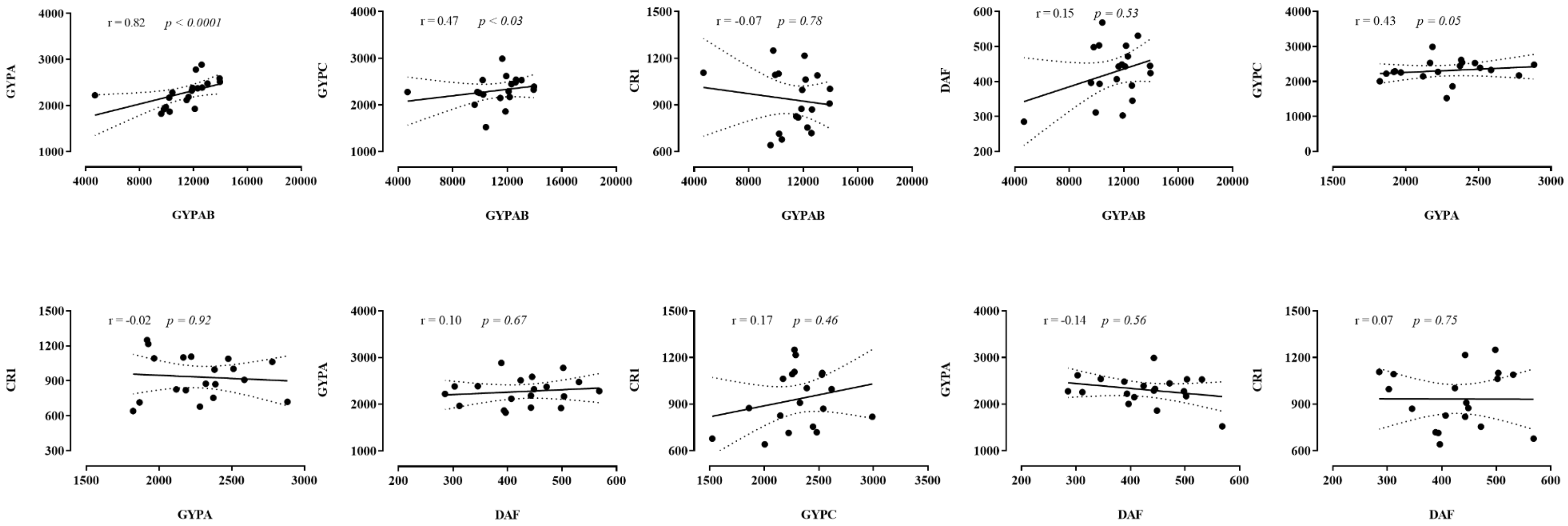

Figure S2: Relationship between erythrocyte receptor densities .The Spearman correlation test was used to assess the relationship between the densities of individual erythrocyte surface receptors. The data were acquired as MFI for the receptor density and the graphs were plotted using Graph Pad Prism v.8.01.

Figure S3

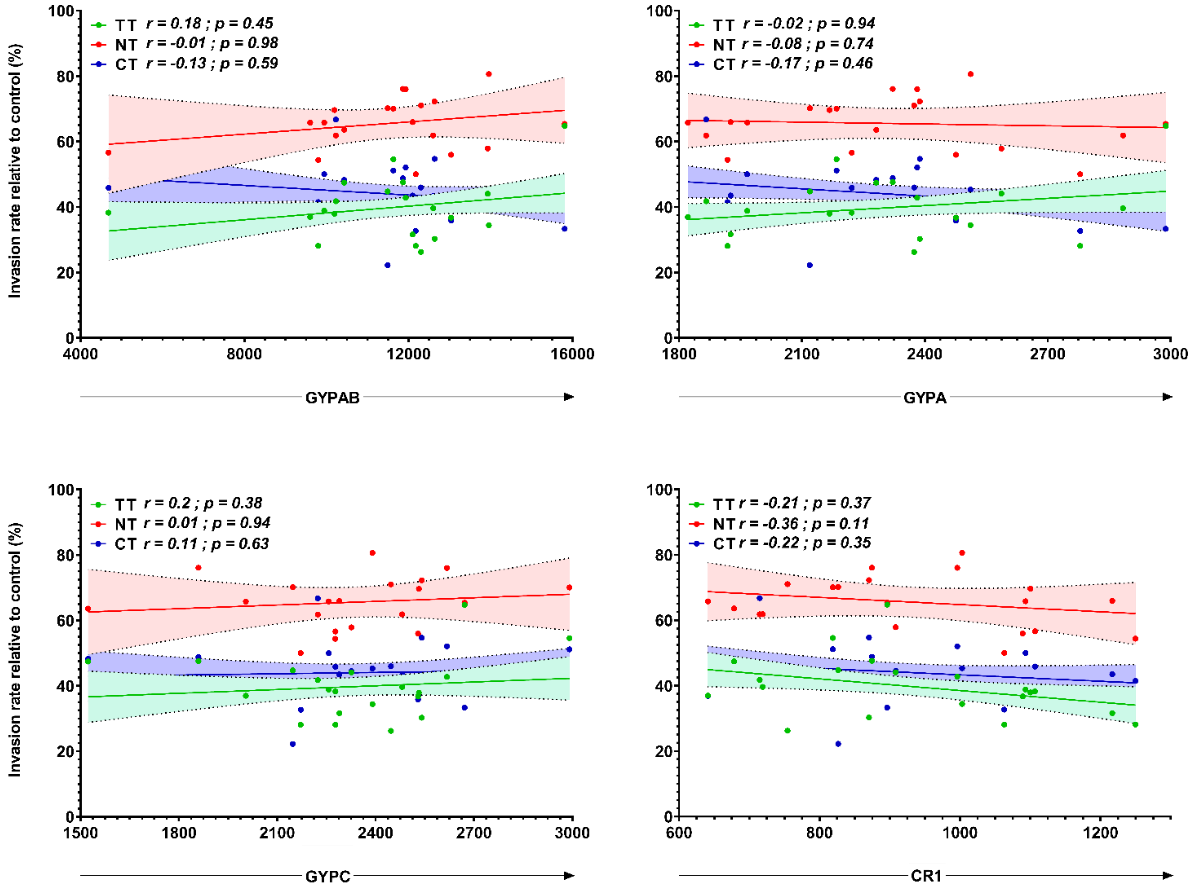

Figure S3: Correlation of receptor density with invasion efficiency into enzyme-treated erythrocytes. The Spearman correlation test was used to assess the relationship between the density of individual erythrocyte surface receptors (X-axis) and the invasion efficiency following enzyme treatment of the same erythrocyte (Y-axis). The data were acquired as MFI for the receptor density and percent parasitemia relative to untreated control erythrocytes for the invasion efficiency and the graphs were plotted using Graph Pad Prism v.8.01.

Figure S4

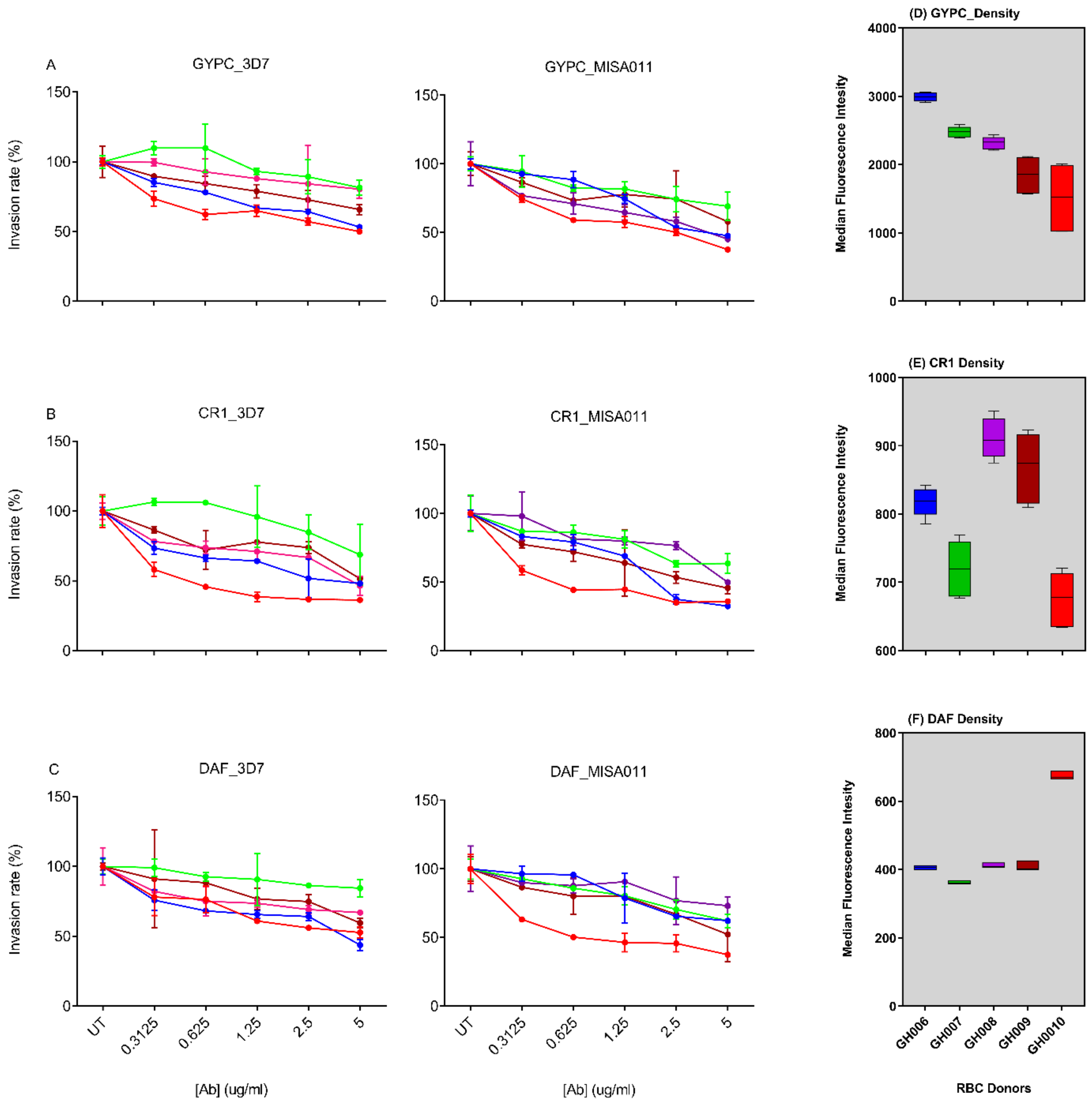

Figure S4: Antibody-dependent invasion inhibition assays in donor erythrocytes with different levels of surface antigens. Schizont-infected erythrocytes were co-incubated with antibody-sensitized uninfected erythrocytes from donors expressing different levels of erythrocyte receptors. The parasites' DNA was labelled with Hoechst 33342, 18-24 hours' post-incubation and the resulting parasitemia was quantified by flow cytometry. For each donor, the parasitemia in the corresponding mock-treated erythrocytes was used to ascertain the antibody-dependent invasion inhibitory, following normalization using a single erythrocyte donor (also used for the parasites *in vitro* culturing). A-C represent the antibody-dependent invasion inhibition for two different *P. falciparum* strains, 3D7 and MISA011. D-F represent the differential expression of erythrocyte receptors assessed in A-C.
